# Push-pull driving of the Central America Forearc in the context of the Cocos-Caribbean-North America triple junction

**DOI:** 10.1038/s41598-019-47617-3

**Published:** 2019-08-01

**Authors:** José A. Álvarez-Gómez, Alejandra Staller Vázquez, José J. Martínez-Díaz, Carolina Canora, Jorge Alonso-Henar, Juan M. Insua-Arévalo, Marta Béjar-Pizarro

**Affiliations:** 1 Department of Geodynamics, Stratigraphy and Paleontology, Faculty of Geology, Complutense University of Madrid, José Antonio Novais, 12, 28040 Madrid, Spain; 2 Dpto de Ingeniería Topográfica y Cartografía, ETSI Topografía, Geodesia y Cartografía, Universidad Politécnica de Madrid, 28031 Madrid, Spain; 3 IGEO Geosciences Institute, Severo Ochoa, 7, 28040 Madrid, Spain; 4 Department of Geology and Geochemistry, Science Faculty, Universidad Autónoma de Madrid, Francisco Tomas y Valiente, 7, 28049 Madrid, Spain; 5 Geohazards InSAR Laboratory and Modelling Group, Geoscience Research Department, Geological Survey of Spain (IGME), Alenza 1, 28003 Madrid, Spain

**Keywords:** Geodynamics, Tectonics

## Abstract

Different kinematic models have been proposed for the triple junction between the North American, Cocos and Caribbean plates. The two most commonly accepted hypotheses on its driving mechanism are (a) the North American drag of the forearc and (b) the Cocos Ridge subduction push. We present an updated GPS velocity field which is analyzed together with earthquake focal mechanisms and regional relief. The two hypotheses have been used to make kinematic predictions that are tested against the available data. An obliquity analysis is also presented to discuss the potential role of slip partitioning as driving mechanism. The North American drag model presents a better fit to the observations, although the Cocos Ridge push model explains the data in Costa Rica and Southern Nicaragua. Both mechanisms must be active, being the driving of the Central American forearc towards the NW analogous to a push-pull train. The forearc sliver moves towards the west-northwest at a rate of 12–14 mm/yr, being pinned to the North American plate in Chiapas and western Guatemala, where the strike-slip motion on the volcanic arc must be very small.

## Introduction

After the establishment of plate tectonics as a paradigm of geology throughout the 1960s, in the 1970s numerous works attempted to explain tectonics in this new theoretical framework. Different kinematic models were proposed to explain the motion of the forearc sliver along the Cocos-Caribbean subduction, in the framework of the triple junction between the North American, Cocos and Caribbean plates, and particularly for the north of Central America. In one of the first proposals it is suggested that the relative drift of the Caribbean plate to the east gave rise to the formation of N-S grabens in Honduras while southern Guatemala and western Honduras remained pinned to North America^1^. This basic model, which did not consider the existence of a forearc sliver yet, was refined, suggesting the existence of a weakness zone along the Volcanic Arc that facilitates the displacement of a forearc sliver dragged laterally, or pulled, by the North American plate motion towards the Northwest^2,3^. These works define the basis of the dragging hypothesis (DH) and the deformation in the trailing edge of the Caribbean plate^4,5^. The higher South America – North America convergence towards the west has been also proposed as mechanism for the Caribbean – North America pinning on its western edge and the relative extrusion of the Caribbean Plate towards the east^6,7^.

As an alternative model to explain the north-westward drift of the forearc sliver the slip partitioning in the subduction was proposed^8^. This model of slip partitioning has been refuted as the subduction interface is not coupled enough to transmit the necessary forces to drive the upper plate forearc^4,9–12^. In addition, it has an insufficient obliquity angle to generate the partition^13^, although it will be discussed below. Towards the southeast of the forearc, in southern Nicaragua and Costa Rica, the GPS vectors show a centrifugal arrangement in front of the subduction of the Cocos Ridge^14^. Some authors suggested the hypothesis that it is the subduction of the Cocos Ridge acting as an indentor, in combination with some partitioning due to a higher subduction interface coupling offshore northern Costa Rica^15,16^, the responsible for the transmission of the necessary forces to the forearc sliver, producing its escape towards the NW (pushing hypothesis, PH)^14,17^.

To date most works on the kinematics of Central America have been focused on northern Central America or on the Cocos Ridge subduction on Costa Rica and southern Nicaragua. In northern Central America the continuity of the forearc sliver from Costa Rica to Chiapas, with the Motagua – Chixoy – Polochic fault system not connected to the Middle America Trench, has been discussed frequently in the context of a diffuse triple or quadruple joint^18–23^.

This paper is a comprehensive view of the Central America forearc kinematics from Chiapas to Costa Rica considering the most commonly accepted hypotheses on its driving mechanism (North American Drag or pull, DH vs Cocos Ridge Push, PH), although also discussing the potential role of the slip partitioning at the subduction interface. We analyze an updated GPS velocity field, earthquake focal mechanisms, regional relief and plate motion obliquity in the subduction. In the view of the available data we propose a coeval push-pull driving mechanism for the motion of the Central America forearc in the context of the North American – Caribbean – Cocos triple junction.

## Implications of Kinematic Models

**Figure 1:**
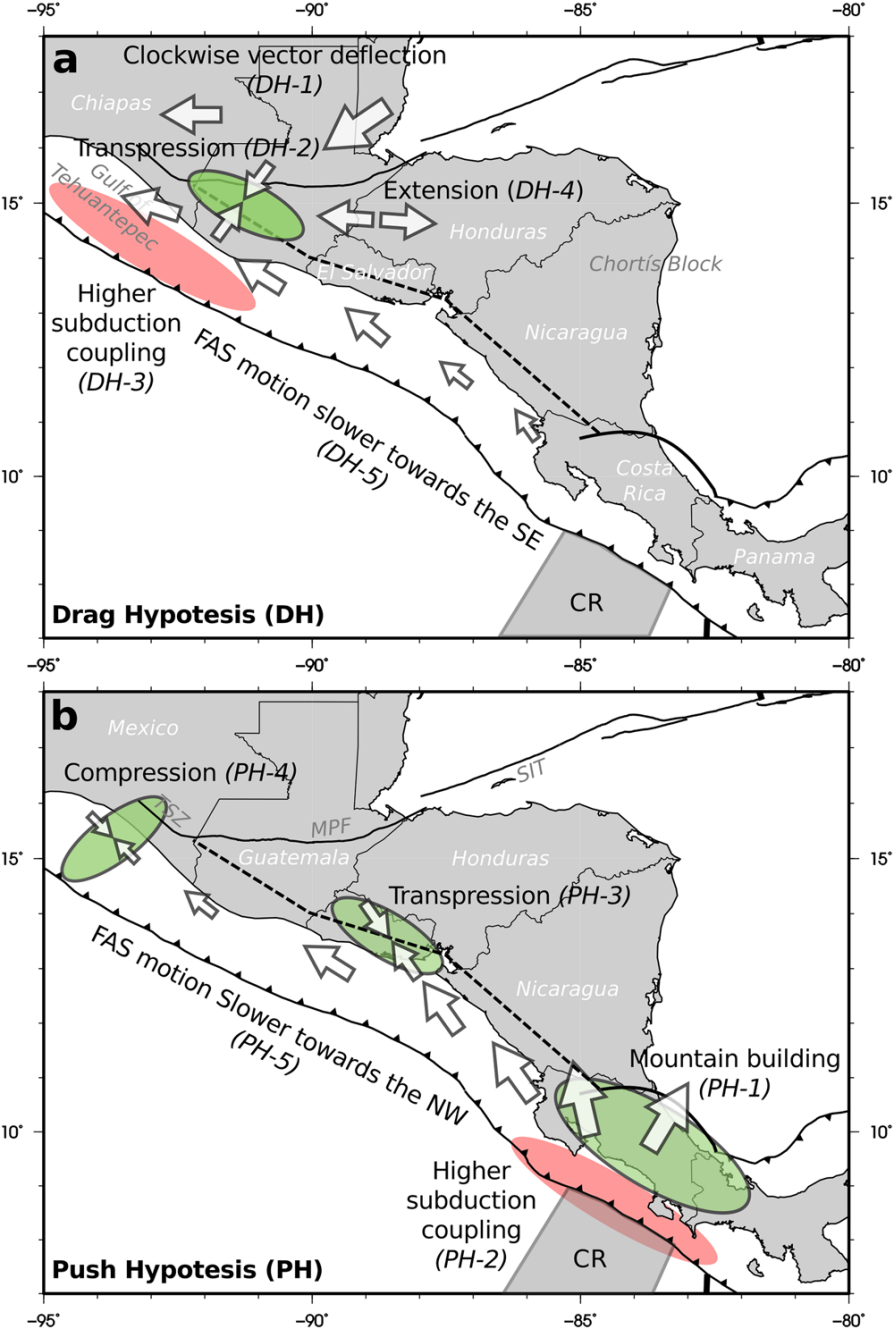
Sketches of the tectonic implications of the discussed kinematic models. (**a**) Lateral drag-based model. The motion of the North American Plate towards the west drags and pulls the forearc sliver. (**b**) Push-based model. The subduction of the Cocos Ridge towards the north-east pushes the forearc sliver to the north-west. In green are shaded the areas where shortening and uplifting are expected. In red are shaded areas where the subduction interface should present higher coupling. The arrows show the predicted motion of the blocks with its size qualitatively proportional to the velocity. CR, Cocos Ridge; TSZ, Tonala Shear Zone; MPF, Motagua – Polochic Fault; SIT, Swan Island Transform. The software package GMT^94^ (version 5.4) has been used to produce the figure.

The two main hypotheses described above, and the slip-partitioning as is discussed below, explain different observations throughout Central America, but can be used also to make kinematic predictions which can be contrasted with existing data (Fig. 1). To make these predictions we have used the results of several models published in the last decade^4,5,14,17,24^. In the case of the drag hypothesis (DH), also described as deformation in the trailing edge of the Chortís block depending on the fixed reference plate (Fig. 1a), it should be expected:

(DH1) The North American plate is moving towards the SW for a Caribbean plate reference frame, this motion is opposed by the Cocos plate motion towards the NNE in the subduction in Chiapas and western Guatemala where both blocks interact. The forearc in this area is then pinned between both motions. Deflection of the plate motion vectors direction in the North American plate from SW to W is expected, accompanied by a deflection from NW to W in the forearc motion vectors.
(DH2) The pinning of the forearc mentioned above must produce compressive or transpressive deformation in the Chiapas area and southwestern Guatemala. The motion of the vectors on both sides of the forearc involves horizontal shortening and, depending on the obliquity of the vectors, horizontal shearing.
(DH3) Increased coupling in the subduction zone in Chiapas and Guatemala is expected due to the North American plate motion towards the Cocos Plate. The motion of the upper plate in a subduction is a key factor controlling the coupling of the interface^25^; also producing upper plate shortening, which has been shown as a controlling factor for the generation of giant earthquakes^26^.
(DH4) E-W extension on the trailing edge of the Chortis block. If we consider the eastward motion of the Chortis Block, for a fixed North American plate, its trailing edge is stretched and deformed internally as its western tip is pinned to the North American plate. The same effect can be described for a fixed Caribbean plate, where the previously described pinning of the Chortis Block in the area of Guatemala is dragging towards the west the western edge of the block, stretching it.
(DH5) If the driving mechanism is the dragging of the forearc in North America, for a fixed Caribbean plate, then the velocities in the forearc should diminish from NW to SE due to elasto-plastic internal deformation, as has been described in other forearcs and tectonic settings^27–29^. A gradient in the forearc sliver velocity decreasing from NW to SE should be observable in the GPS velocity field.

The Cocos Ridge push hypothesis (PH), on the other hand, predicts

(PH1) Uplifting and compression in Costa Rica. The subduction of aseismic ridges and submarine relief usually induces uplifting in the overriding plate^30–33^ and if the Cocos Ridge acts as an indenter^14^, arc-normal shortening.
(PH2) The submarine relief and roughness is usually related to the presence of small patches of higher coupling in the subduction interface^34–37^. Consequently the roughness of the Cocos Plate and the Cocos Ridge subducting at Costa Rica influence the subduction behavior^38^, predicting a higher subduction interface coupling.
(PH3) If the Cocos Ridge subduction is the responsible of the forearc northwestward displacement, acting as an escaping block moving parallel to the volcanic arc in Nicaragua, then transpressive deformation is expected in the left bend produced in the volcanic arc in El Salvador. This mechanical prediction, in addition to be a geometrical structural requirement^39^, can also be deduced from the LaFemina^14^ kinematic model.
(PH4) Towards the northwest the escaping forearc sliver must be colliding with the North American plate, which should be reflected as SE-NW forearc shortening^40,41^.
(PH5) Analogously with the DH5 prediction, if the driving mechanism of the forearc sliver motion is located at its southeastern tip, then a decrease in the velocity field of the forearc sliver from the SE to NW should be observable.

These predictions are summarized in Table 1.

**Table 1:** Summary of the kinematic predictions extracted from both models discussed in the text.

	ID	Prediction	Relief	Seismicity	GPS
Drag	*DH-1*	*Clockwise rotation of velocity vectors*			✓
	*DH-2*	*Transpressive deformation at Chiapas and SW Guatemala*	✓	✓	✓
	*DH-3*	*Higher coupling in Chiapas-Guatemala subduction*		✓	✓
	*DH-4*	*E-W extension at Honduras*	✓	✓	✓
	*DH-5*	*Slower forearc motion at SE*			✓✗
Push	*PH-1*	*Mountain Building and shortening at Costa Rica*	✓	✓	✓
	*PH-2*	*Higher coupling at Costa Rica subduction*		✓	✓
	*PH-3*	*Transpression at El Salvador*	✗	✗	✗
	*PH-4*	*SE-NW compression at Tehuantepec*	✗	✗	
	*PH-5*	*Slower forearc motion at NW*			✓✗

Check mark indicates supported, x mark indicates rebated and ambiguous is indicated by both signs at the same time. Empty cells indicates that the available data are not suitable to check the prediction.

The role of the slip partitioning as a primary or secondary driving mechanism will be discussed below with an obliquity analysis taking into account the interface earthquake slip vectors.

## The Data

To test the predictions of both hypotheses we analyze relief data, seismicity and GPS velocity fields. We choose these data sources because they cover different time scales and tectonic processes. The relief shows the recent tectonic configuration and regional scale processes, particularly the vertical motions, but also the structural main trends. The seismicity reflects the brittle deformation of the crust in the last decades and is commonly used as a proxy to the state of stress on the lithosphere. The updated GPS velocity field shows the present tectonic blocks motion and strain gradients, including elastic and plastic strains.

### Relief

The development of relief is strongly coupled to large-scale feedback involving the interplay of climate, erosion and tectonics^42^. The tectonic component in this context is understood as the set of processes generating bedrock uplift, including the isostatic uplift^43^. Additionally, volcanic processes play also an important role, specially in the local relief. Taking into account its complexity the relief allows us to identify the areas where there have been recent tectonic uplifting processes. This serves as a proxy to check the predictions related to the tectonic uplift linked to compressive and transpressive deformations.

**Figure 2:**
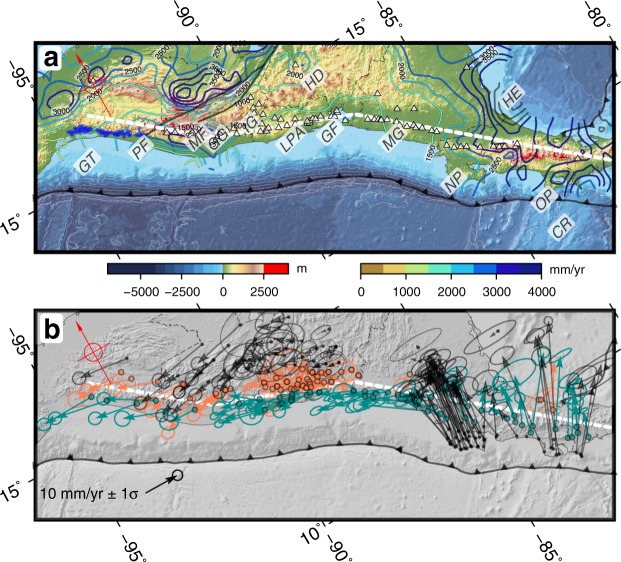
Maps of regional data compiled. (**a**) Topographic and geologic data. The digital elevation model shows the relief from GEBCO_2014 Grid (version 20150318, www.gebco.net). In bright red are highlighted the areas with elevations higher than 2500 meters. In blue is shown the Miocene Chiapas Arc plutons^90^. The white triangles show the active volcanoes (Global Volcanism Program, 2013). The contours show the mean annual precipitation^93^. GT, Gulf of Tehuantepec; PF, Polochic Fault; MF, Motagua Fault; GG, Guatemala Graben; IG, Ipala Graben; LPA, Lempa Pull-Apart; GF, Gulf of Fonseca; MG, Managua Graben; NP, Nicoya Peninsula; OP, Osa Peninsula; HE, Hess Escarpment; HD, Honduras Depression; CR, Cocos Ridge. (**b**) Geodetic data. The arrows show the compiled GPS data unified to the same reference frame^5,11,12,17,24,56^, in blue and orange vectors located to the south and to the north (on a 130 km width band) of the Central America Volcanic Arc respectively, as shown in Fig. 3. The velocities are relative to a fixed Caribbean plate using the ITRF2005 reference frame. The ellipses show the 1σ uncertainty. The thick dashed white line shows the approximate position of the Central America Volcanic Arc used to project the GPS data in Fig. 3. The software package GMT^94^ (version 5.4) has been used to produce the figure.

The topo-bathymetric data used is from the GEBCO_2014 Grid (version 20150318, www.gebco.net), which uses SRTM30 global data for the topography with a resolution of 30″. In Fig. 2a the areas with greater relief have been marked in red (height > 2500 m), being located in Costa Rica and Southwest Guatemala (PH-1 and DH-2 predictions).

The amount of precipitation can be considered a proxy for the denudation in a region^44^. The denudation competes with the tectonic uplift to reach a steady-state topography of equilibrium^45^. As is evident from Fig. 2a there is no negative correlation between the annual precipitation rate and the relief in Central America (i.e. the lowlands are not related to higher precipitation rate) and consequently the observed uplift must be the result of tectonic processes rather than climatic, although we cannot rule out complex interactions between erosion rate, mountain building and geomorphic forms as discussed in other mountain ranges^46–48^.

**Figure 3:**
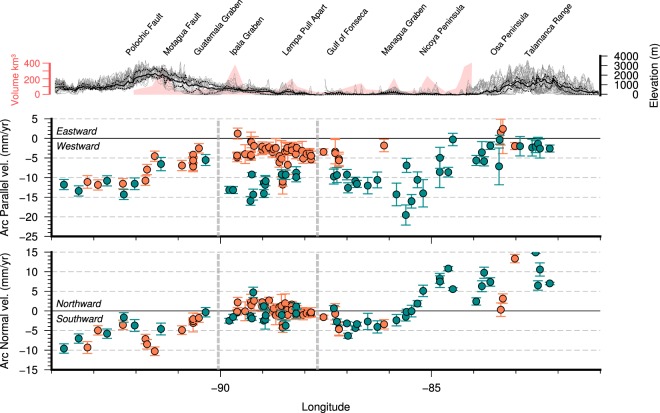
GPS velocity profiles showing the Arc-Parallel and Arc-Normal components of the stations located to the south (blue) and to the north (orange) of the Volcanic Arc mean line shown on Fig. 2 on a band of 130 km width. The error bars correspond to 1σ. See Fig. 2 caption for details on GPS data origin. In the upper part of the figure is shown the topographic swath profile with the main tectonic features as spatial reference and in light red the eruptive volume of each volcanic center along the arc^50^. The software package GMT^94^ (version 5.4) has been used to produce the figure.

Although the volcanic activity has the potential to influence greatly the local relief, in the regional context of Central America seems to have little impact. An example is the volcanic activity in Nicaragua, which has been proposed as the more active section of the volcanic arc, or at least equally active as the rest of the arc^18^, and shows little influence on the total relief of the Nicaraguan sector of the arc (Fig. 2a). Similarly the topographic swath profile shown in Fig. 3 is not directly related to the volcanic production rates estimated for the arc^49,50^.

In Costa Rica the Cocos Ridge subduction has produced the uplift of the Cordillera de Talamanca in recent times^51^, by means of tectonic shortening^52,53^ or isostatic compensation^54^. In Guatemala and Chiapas a recent tectonic uplift caused by the forearc – North America shortening has been also described; in relation with the diffuse triple junction and the forearc sliver pinning to North America^23^, and with the complex fault interactions of the triple joint in a zipper model^19^.

The relief also allows us to identify recent tectonic structuring, such as the presence of N-S grabens in Honduras (DH-4 prediction) and NW-SE folds in Chiapas (DH-2 prediction). The N-S grabens in Honduras are the result of the recent extensional deformation of an uplifted Miocene ignimbritic plateau, being the uplift produced by mantle upwelling after the Cocos subducted slab detachment^55^. The folding in Chiapas is directly related to the tectonic uplift caused by the North American plate – forearc sliver convergence^23^, maybe as part of a compressional jog or restraining right stepover^21^.

### GPS

We have generated a GPS velocity field referencing to a common system the data published by different authors^5,11,12,17,24,56^. Because these velocity fields were in a different global reference frames (ITRF2000, ITRF2005, etc.) we have recalculated them to a fixed Caribbean plate using different poles depending on the initial frame (for details on the original data processing please check the referenced works). The use of the Caribbean plate as fixed is the most used reference in the area and the most suitable to observe the displacement of the forearc with respect to the back-arc (a velocity field referenced to a fixed North American plate is presented as supplementary material). The obtained velocity field is consistent (Fig. 2b), although there may be subtle differences between the results obtained by the different authors in common stations in the different works. These differences are within the measurement error and therefore the velocity field can be considered as representative of the interseismic deformation in the zone. Recently a new GPS velocity field for northern Central America has been published integrating and processing jointly data previously processed separately^57^. The GPS field presented in this work is equivalent and presents the same general picture, although with higher uncertainties. We have projected these data on a line defining the approximated trend of the volcanic arc and obtained the parallel and normal components (Fig. 3).

In the velocity field, an important trench-normal component can be observed in the Nicoya and Osa peninsulas in Costa Rica, a product of the higher subduction interface coupling and the Cocos Ridge subduction^14^. This subduction generates an important shortening in the mountain range of Costa Rica (PH-1 prediction). In Guatemala and Chiapas there is also an important trench-normal component, especially to the north of the volcanic arc, but directed towards the west instead of to the north as in Costa Rica. This component is consistent with the motion of the North American plate towards the Cocos Plate in the Gulf of Tehuantepec, generating also a deflection of the velocity vectors (DH-1 prediction). From Costa Rica to Guatemala, the trench-normal component is minimal, which implies very low or zero subduction interface coupling, as has already been described in many works^4–6,10,12,24^.

In the triangle formed by the Motagua fault, the Volcanic Arc and the Honduras depression it is evident an increase in velocities from east to west, consistent with a significant internal deformation^5^. On the other side of the Motagua fault the GPS vectors clearly mark the displacement of the North American plate towards the west, being the change very abrupt. Towards the south the gradient is less pronounced although it is clearly located in the Volcanic Arc, with an increase of the velocity towards the west. The GPS vectors in the North American plate and in the forearc in Chiapas show very similar trends and velocities^24^.

The mentioned arc-normal component in Costa Rica becomes an arc-parallel displacement towards the NW (Fig. 3). The arc-normal component changes from positive in Costa Rica to negative in Nicaragua, while the arc-parallel velocity decreases from 20 mm/yr to about 10 mm/yr near the Gulf of Fonseca (PH-5 Prediction). This arc-parallel component, however, increases again from the Gulf of Fonseca (10 mm/yr) to the Ipala Graben area (15 mm/yr) (DH-5 prediction). The normal component is equal on both sides of the volcanic arc along the whole profile except in two zones, between the Motagua fault and the Graben of Guatemala, where there seems to be some shortening (although based only on one station), and between the Ipala Graben and the Gulf of Fonseca, where there appears to be an extension of up to 4 mm/yr^56^.

The arc-parallel component of velocity shows three sections with different characteristics (Fig. 3). Towards the west the data shows the same trend to the north and to the south of the volcanic arc. This is consistent with the absence of horizontal shearing from Chiapas to the Ipala graben. This would imply that the Jalpatagua fault can only be active on its eastern tip, eastward of the Guatemala Graben. From the Ipala Graben to the Gulf of Fonseca, there is a decrease in the velocity difference between the north and south stations, from 12–14 mm/yr to 3–5 mm/yr (DH-5 prediction). It should be noted, however, that it is possible that the installed GPS network is not adequately recording the deformation in the easternmost part of El Salvador, where there is a distribution of the deformation with active structures towards the Pacific coast, with an estimated E-W extensional deformation of around 4 mm/yr in the Jucuarán-Intipucá coastal range^58^. From the Gulf of Fonseca towards the east the arc-parallel component increases reaching a maximum in the area of the Nicoya Peninsula (PH-5 prediction). This arc-parallel motion becomes a north-directed normal component towards the east of the profile as a consequence of the centrifugal arrangement of the GPS velocity vectors (Fig. 2b).

### Seismicity

**Figure 4:**
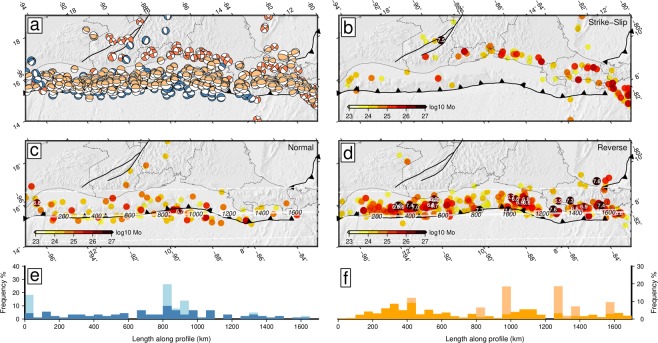
Earthquake focal mechanisms from the Global CMT catalog^59^ with hypocentral depths shallower than 25 km to highlight the cortical deformation. (**a**) Beach-balls of the focal mechanisms with the shading showing the type of rupture; blue: normal faulting; red: strike-slip faulting; yellow: reverse faulting. (**b**–**d**) Maps of released seismic moment by the type of rupture in dyn·cm^−1^, (**b**) strike-slip, (**c**) normal, (**d**) reverse. In (**c**,**d**) the profile and the buffer to project the subduction related seismicity is shown with labels of length along the profile in km. (**e**) Histogram of proportion of subduction shallow normal earthquakes projected on the profile shown in (**c**). The light blue bars show the proportion weighted by the seismic moment released. (**f**) Histogram of proportion of subduction shallow reverse earthquakes projected on the profile shown in (**d**). The light orange bars show the proportion weighted by the seismic moment released. The software package GMT^94^ (version 5.4) has been used to produce the figure.

**Figure 5:**
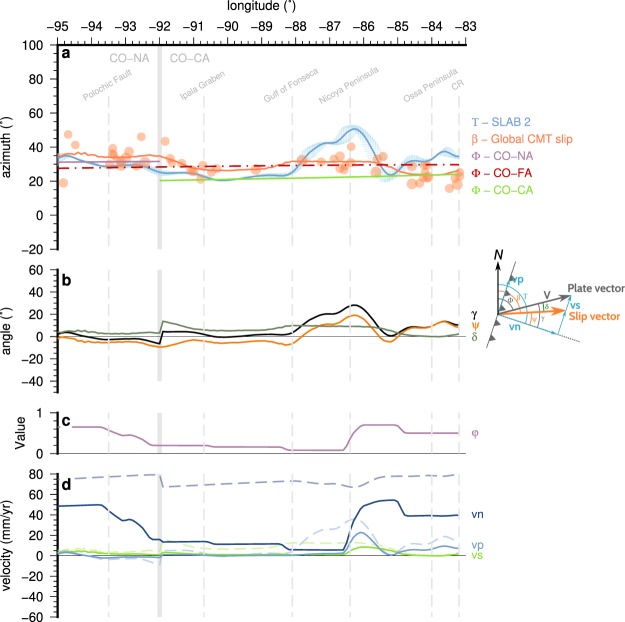
Obliquity and partitioning analysis of the Cocos-Caribbean and Cocos-North American subduction at Central America. (**a**) Azimuth of the different slip vectors on the subduction interface. The blue shaded area, and the thick blue line, represents the azimuth of the subduction interface normal (Τ) on the upper 30 km from the Slab2 model^71^. The plate motion vector azimuth (Φ) has been computed with respect to a fixed North American plate for longitudes between −95 and −92 (purple line), to a fixed Caribbean plate for longitudes between −92 and −83 (green line), and to a fixed forearc sliver for the whole area (red dashed line). Finally, the back azimuth (β) of the slip vector from the northeast dipping nodal planes of the reverse earthquakes are shown as orange circles. We have selected the reverse events near the trench shallower than 40 km with M_W_ >5.9 from the Global CMT^59^ focal mechanisms catalog. These events can be considered representative of the relative plate motions^72^. A trend line using a gaussian filter has been obtained to be used in the following calculations (thick orange line). The size of the circle is proportional to the earthquake magnitude. (**b**) Obliquity defined as the angle between the trench normal and the motion vector. γ is the plate convergence obliquity. Ψ is the focal mechanisms slip vector obliquity (see inset for details), δ is the difference between both angles, the residual slip angle. (**c**) Values of the subduction interface coupling (φ). (**d**) Velocities vp and vn are the margin parallel and margin normal components of the plate motion vector. vs is the predicted forearc sliver slip rate relative to the upper plate. The dotted line show the rates obtained assuming a fully coupled subduction interface. The solid lines show the actual rates taking into account the subduction interface coupling shown in (**c**). The software package GMT^94^ (version 5.4) has been used to produce the figure.

The seismicity is a reflection of the brittle deformation of the lithosphere, either internal deformation of tectonic blocks, giving rise to seismicity of moderate magnitude, or deformation associated to the limits of these, giving rise to major earthquakes. In Fig. 4 we present the shallow seismicity (<25 km depth) of the Global CMT catalog^59^, reflecting the active processes of cortical deformation. This catalog spans from 1976 to the present and is complete for magnitudes over Mw 5.3 and smaller for the last decades^59^. The smaller events can be affected by local stress perturbations, but the greatest show a good coherence and low variability of its slip vectors (Fig. 5) and can be considered representative of the plate interactions.

We have classified the events by their type of rupture in normal (blue compressive quadrants), reverse (orange compressive quadrants) and strike-slip (red compressive quadrants) (Fig. 4a). The strike-slip events delineates the major transcurrent structures: the Caribbean – North American plate boundary, with the 1976 Mw 7.5 Guatemala earthquake (Fig. 4b); the volcanic arc deformation zone spanning from Guatemala to Costa Rica; and the Panama Fracture Zone, in the southeastern tip of the mapped region. In addition to these event lineations other strike-slip earthquakes are present also in the Gulf of Tehuantepec and the Hess escarpment.

Two main families of normal events can be distinguished. One at the western end of the Chortís block, with planes of N-S approximate orientation (DH-4 prediction), and another forming a band along the trench with trench-parallel nodal planes, characteristic of slab bending processes. We have projected the shallow normal faulting events along the trench (in a 300 km buffer) (Fig. 4c) and computed a frequency histogram in bins of 50 km (the light blue bars are events weighted by its seismic moment) (Fig. 4e). The maximum frequency of bending-related normal events is located offshore El Salvador and Northern Nicaragua.

We found the reverse faulting events delineating the subduction (Fig. 4c), with major clusters of events, probably related to higher seismic coupling, in the zones of Chiapas-Guatemala (DH-3 prediction) and central Costa Rica (PH-2 prediction). As in the normal fault events, we have projected the shallow reverse fault events along the trench (Fig. 4f). Three areas with higher frequencies can be distinguished: offshore Chiapas and Guatemala an elongated cluster of events with maximum magnitude Mw 7.4^60^; offshore Nicaragua a cluster of events with maximum magnitude Mw 7.6 corresponding to the 1992 Nicaragua tsunami earthquake and aftershocks^61^; and along the coast of Costa Rica, with a higher frequency of major earthquakes. Moreover the characteristics of the thrust events are different along the subduction interface. The Nicaragua 1992 and El Salvador 2012 events present characteristics typical of tsunami earthquakes while the Guatemala 2012 and Costa Rica 2012 events are typical subduction interface earthquakes^60^.

In addition to these events, others appear in a smaller proportion in the continental crust of Chiapas (DH-2 prediction) and in the Caribbean margin of Costa Rica (PH-1 prediction); being a reflection of internal deformation by tectonic shortening.

## Discussion and Conclusions

### Obliquity and slip partitioning

Slip partitioning was defined in subduction zones with oblique convergence showing a strike-slip zone parallel to the trench^62–64^; since then it is frequently advocated as driving mechanism on every subduction zone with a forearc sliver, and the Middle America Trench at Central America is no exception^8,65^. Despite having been discarded in the region on several occasions due to the low coupling of the subduction interface^4,5,9–12^, one of the requirements for it to be an efficient mechanism, oblique subduction is still sometimes referenced as the cause of displacement of the forearc sliver in Central America^66–69^. In order to check the validity of the obliquity and slip partitioning as driving mechanism we have performed an obliquity analysis of the Middle America Trench throughout Central America.

When a plate subducts obliquely its motion vector can be absorbed decoupling the trench normal component, which is usually absorbed as reverse faulting into the subduction interface, and trench parallel component taken up by strike-slip on a transcurrent fault within the overriding plate^62^. This slip partitioning process is characterized by the azimuths of the subducting plate motion vector (Φ), the arc-normal (Τ) and the reverse faulting earthquakes slip vector (β)^70^.

In Fig. 5a the different parameter azimuths are shown following the notation represented in the vectors sketch^70^. The blue shaded area, and the thick blue line, represents the azimuth of the subduction interface normal (Τ) on the upper 30 km from the Slab2 model^71^. The plate motion vector azimuth (Φ) has been computed with respect to a fixed North American plate for longitudes between −95 and −92 (purple line in Fig. 5a), to a fixed Caribbean plate for longitudes between −92 and −83 (green line in Fig. 5a), and to a fixed forearc sliver for the whole area (red dashed line in Fig. 5a). Finally, the back azimuth (β) of the slip vector from the northeast dipping nodal planes of the reverse earthquakes are shown as orange circles. We have selected the reverse events near the trench shallower than 40 km with M_W_ > 5.9 from the Global CMT^59^ focal mechanisms. These events can be considered representative of the relative plate motions^72^. A trend line using a gaussian filter has been obtained to be used in the following calculations (thick orange line). From these azimuths the angles between them are computed (Fig. 5b) defining the plate motion obliquity, γ = Τ − Φ; the slip vector obliquity, ψ = Τ − β; and the slip vector residual, δ = γ − ψ = β − Φ. Using the subduction interface coupling (φ) (Fig. 5c) we obtain the different potential velocities of the forearc sliver (Fig. 5d) assuming a fully coupled subduction interface (dashed lines) or the average coupling modeled for the subduction interface^12,14,17,24,57^.

The trench-normal azimuths show values between N20° and N50°, with slight variations from the Gulf of Tehuantepec (longitude −95) to the Gulf of Fonseca, reaching the value of N20° at El Salvador. From the Gulf of Fonseca an abrupt change in the direction of the trench is shown, reaching values of N50° at the Nicoya Peninsula. Eastward of the Nicoya Peninsula the trench-normal adopt values around N30°. The azimuth of the subducting plate motion vector in Chiapas and western Guatemala, for a fixed North American plate^73^, has values between N31.6° and N31.2° while in the rest of the trench, for a fixed Caribbean plate^73^, the values are between N20.4° and N24.1°. If we compute this motion vector for a fixed forearc sliver^57^ the values are between N28° and N30°. The earthquakes slip vector back azimuth define three zones along the trench. In the subduction in Chiapas and western Guatemala the back azimuth present values ~N35°, from the longitude −92 towards the Ipala graben longitude there is a gradual transition to values of ~N26° and then again a slight increment towards values of ~N31° in the area of the Nicoya Peninsula. In the subduction of the Cocos Ridge, in the area of the Ossa Peninsula, the values diminish to ~N23°.

From the vector sketch shown in Fig. 5b can be clearly seen how the potential forearc slip component (vs) is directly related to the slip vector residual (δ). As the back azimuth of the earthquake slip vector (orange line in Fig. 5a) is always greater than the azimuth of the plate motion vector (purple and green lines in Fig. 5a), the slip vector residual (δ) is positive (dark green line in Fig. 5b). When δ = 0 there is no slip partitioning as the plate motion vector equals the reverse earthquakes back azimuth. Depending on the relation of the angles γ and ψ (Fig. 5b) different partitioning situations may arise.

**Figure 6:**
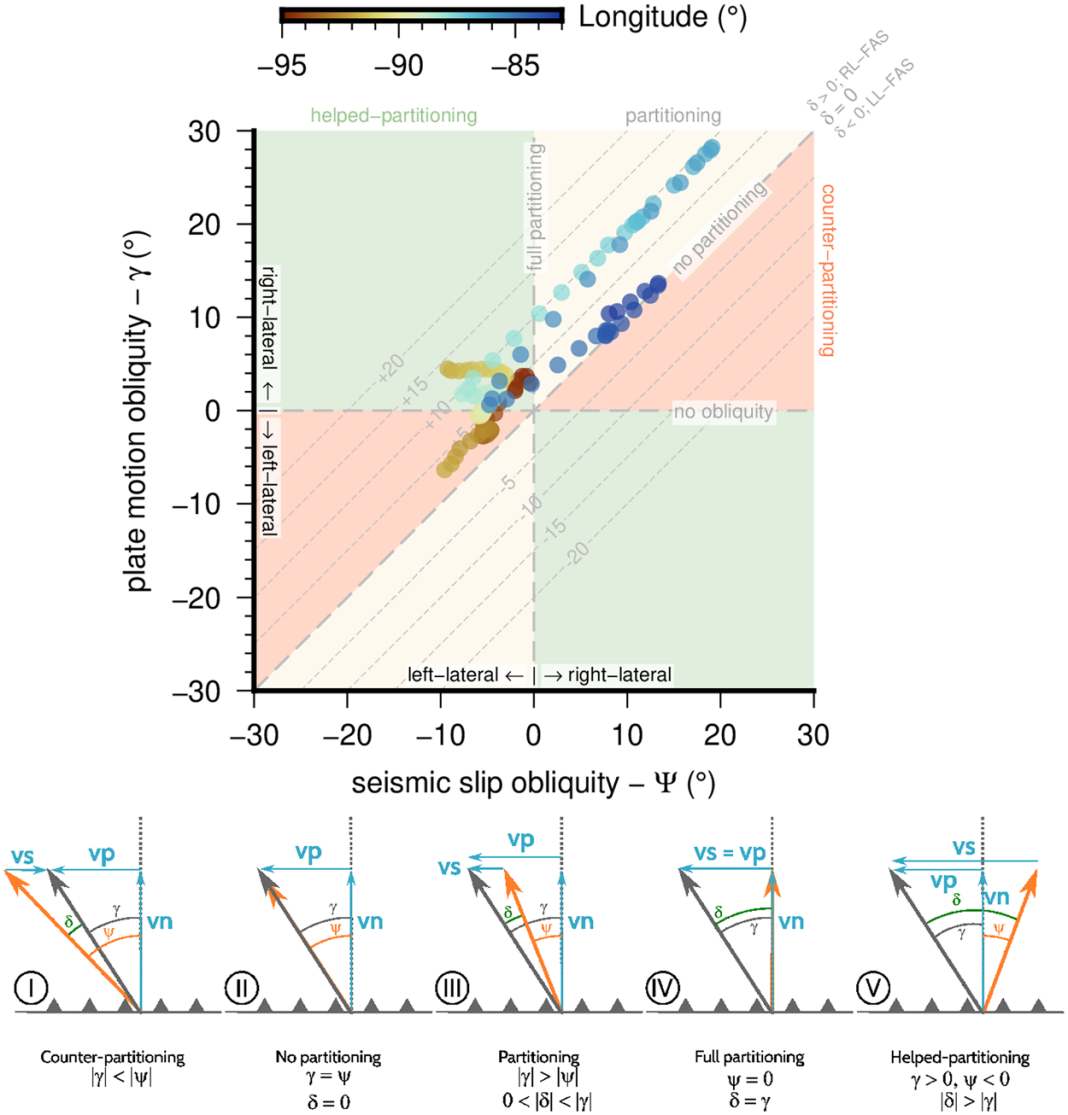
Seismic obliquity (Ψ) versus plate motion obliquity (γ) graph. The shaded areas show the different partitioning situations depending on the relations between Ψ and γ (see bottom diagrams, I–V, of the figure). Yellow, areas where usual partitioning takes place; green, areas where additional external motion is needed to fulfill the angular requirements (helped-partitioning); red, areas where additional external motion counteracting the forearc sliver motion is needed (counter-partitioning). The horizontal line γ = 0 means that partitioning is not possible; the vertical line Ψ = 0 means that partitioning is complete. The diagonal dashed lines show different values of residual angle δ, being positive for right-lateral forearc motion and negative for left-lateral forearc motion. The diagrams at the bottom of the figure show schematically the possible angular relations between vectors. See caption of Fig. 5 for details on the different vectors. The software package GMT^94^ (version 5.4) has been used to produce the figure.

In Fig. 6 a set of diagrams showing the possible relations are shown. Tipically the slip partitioning is described in subduction zones where γ > ψ and the azimuths of the plate motion and slip vectors are both oblique in the same sense from the trench-normal vector^63,64,70,72,74–79^ (situation III in Fig. 6, vs >0). When γ = ψ then δ = 0 and there is no partitioning in the subduction (situation II in Fig. 6, vs = 0). If ψ = 0 then δ = γ and the partition is full (situation IV in Fig. 6, vs = vp). This is because these are the cases in which the slip partitioning is expected. However, when analyzing the angular relationships between the various slip vectors in a complex subduction zone, we observe that these cases represent some of all the possibilities. If γ < ψ then δ < 0 (situation I in Fig. 6, vs < 0) and the motion of the forearc sliver must be opposite to the subduction obliquity. Finally if γ > 0 and ψ < 0 (divergent obliquities) then δ > γ (situation V in Fig. 6, vs > vp) and the motion of the forearc sliver must be faster than that predicted by the obliquity of the plate motion vector. In these two end cases external forces must be present to fulfill the kinematic necessities. We have termed counter-partitioning to the former situation and helped-partitioning to the latter. Although these different situations can be deduced from Fig. 5 we have plotted the γ − ψ pairs along the Central America subduction in Fig. 6. It is noteworthy that this plot is equivalent to the used in previous analyses^64^ but allowing the unexpected negative values of γ and ψ. We have shaded the areas with the different kinematic situations and marked diagonal lines for a range of δ values.

Between longitudes −85° and −83°, in the area of the Osa Peninsula and Cocos Ridge subduction, γ = ψ and δ = 0 (dark blue dots in Fig. 6) which implies that no slip partitioning is taking place in the area. Between longitudes −85.5° and −88°, in the area of the Nicoya Peninsula and Nicaragua, γ > ψ and δ < γ, and consequently the partitioning is possible, although the residual angle δ is small (10°)(light blue dots in Fig. 6). Between the Gulf of Fonseca (~−88°) and the Ipala Graben area (~−90.6), offshore El Salvador, γ = 0, the plate motion vector is normal to the trench, but ψ < 0 and then δ > γ, meaning that the forearc motion must be produced elsewhere (helped-partitioning) (light brown dots in Fig. 6). From the Ipala Graben area to the west there is a transition clearly seen in the earthquake slip vectors (Fig. 5a) with back azimuths changing from N25°E in El Salvador to N40°E in Guatemala. This change is also shown in Fig. 5b, where the obliquity direction change from right-lateral to left-lateral. In Fig. 6 the angular relations for these longitudes (from −92 to −94) are located in the counter-partitioning field, meaning that although the obliquity predicts a left lateral motion of the forearc sliver if it is driven by partitioning, the forearc sliver motion is in fact right lateral (δ > 0), and again, external kinematic causes are needed (brown dots in Fig. 6).

In addition to these obliquity constraints, if we take into account the subduction interface coupling (φ) (Fig. 5c) the maximum arc-parallel predicted velocity components (solid lines in Fig. 5d) are very small, and the sliver velocity (vs) almost negligible, except in the area between Nicoya and Osa peninsulas.

From this obliquity analysis it is clear that the role of the slip partitioning as driving mechanism of the forearc sliver could only be valid locally in central-southern Costa Rica. In order to drive the forearc sliver in the rest of Central America additional external mechanisms are needed.

### Driving mechanism

The drag model presents a better general fit to the observations, although the push model adequately reflects the observations in Costa Rica and Southern Nicaragua. The drag model is able to explain the observations for most of the forearc. Towards the SW it would be the subduction of the Cocos Ridge the responsible for the increase in GPS velocities. The displacement of the Central American forearc towards the NW would be analogous to a Push-pull train with the main locomotive on the head dragging and another locomotive pushing on the tail.

The deformation observed in the GPS velocity field is highly conditioned by singular structures of lithospheric scale such as the Ipala Graben or the Honduras Depression (Fig. 3), which clearly separate different deformation domains^4,5,24,80^. These lithospheric limits are also shown on the topography (Fig. 3) and can be related to the Moho depth variations along the forearc and the volcanic arc^81^; variations on the geochemistry of magmas along the volcanic arc have been also related to lithospheric and subducting slab characteristics^82^. The forearc sliver moves towards the west-northwest with respect to the Caribbean plate at a rate of 12–14 mm/yr, being pinned to the North American plate in Chiapas and western Guatemala. There is no right-lateral strike-slip motion on the relict volcanic arc of Chiapas, where the relative motion between North America and Caribbean plates is accommodated northward by left-lateral faults (e.g. the Tuxtla–Malpaso fault system, the High Sierra fault system)^22,83^. In western Guatemala, according to the our data, the strike-slip motion on the volcanic arc must be very small. These short term scale results support the fact that there is not active forearc sliver west of Guatemala in the North American plate, thus so either the sliver never existed or it is sutured in this zone^19,84^.

The lack of strike-slip activity along the volcanic arc in western Guatemala can be interpreted as a propagation towards the southeast of a tectonic suture on a closing zipper-type triple junction (also called extraction fault)^19,85,86^. In this kind of junctions the strike-slip motion in the closing fault is substituted by a transpressional deformation prior to its definitive suturing^87^.

The Polochic fault intersection with the volcanic arc marks the end of the recent volcanic activity (Fig. 2) which coincides with the end of the forearc sliver. The extinction of the volcanic arc in the Sierra Madre de Chiapas can be explained by the suture of the triple junction zipper model^19^ but have also been related to the change of dip and break off of the subduction slab below Mexico^88,89^. Both processes are not mutually exclusive and could be related.

The Miocene Chiapanecan arc magmatism was active since ca. 12 Ma in the late middle Miocene and it likely continued until ca. 9 Ma^90^. This volcanic arc was affected by left-lateral shearing in the Tonalá shear zone, probably as a continuation of the Motagua – Polochic shear zone^90^, and by arc-normal shortening on a transpressional setting^4,21,91^. These geological observations are well explained by a closing zipper model^19^ presenting similarities with the Eurasia – Arabia – Anatolia triple junction^87,92^. However, alternative explanations for the absence of strike-slip motion in western Guatemala cannot be ruled out.

If there is an eastward displacement of the suture, then there is a transfer of material from the forearc (caribbean plate) to the North American plate. This different behavior of the forearc can be observed in the GPS velocity field. The forearc attached to the North American plate moves with the same trend and velocity of the latter, as it is clearly seen in Chiapas and western Guatemala (Figs 2b and 3).

The different behavior along the forearc should also be observed in the subduction characteristics. The forearc of the North American plate thrusts over the Cocos plate, while the Caribbean forearc slides parallel to the trench. This is reflected in a higher coupling of the subduction interface in Chiapas and western Guatemala^24^, a higher reverse seismicity rate (Fig. 4f) and a different orientation of the rupture slip vectors of the focal mechanisms along the trench (Fig. 5a). In Chiapas and Guatemala the orientation of the rupture slip vectors is close to the orientation of the Cocos – North America convergence^73^ (Fig. 5), the location of this transition coincides with an area of diffuse deformation on the forearc sliver^22^. In southern Costa Rica the orientation of the rupture slip vectors clusters around the orientation of the Cocos – Caribbean convergence^73^ (Fig. 5a). In El Salvador and Northern Nicaragua the orientation seems to be between both, but closer to the Cocos – North America trend and lying exactly on the Cocos-Forarc sliver predicted motion^57^.

**Figure 7:**
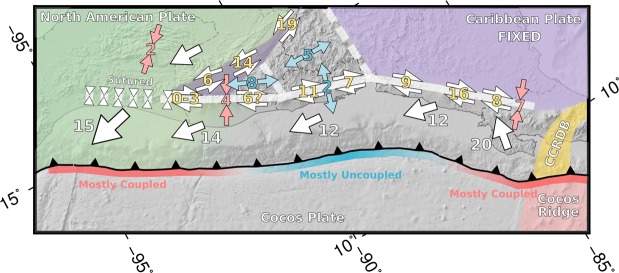
Kinematic model with average velocities of blocks and faults. The numbers show velocities in mm/yr. In orange velocities of mainly strike-slip displacement with the sense of motion marked by the arrows. Extension and shortening rates in blue and red respectively. The white numbers and arrows show the horizontal motion of blocks. The green shaded area correspond to the North American plate, the violet shaded area corresponds to the fixed Caribbean plate, the red shaded area to the Cocos Ridge bathymetric feature and the yellow shaded area to the Central Costa Rica Deformed Belt (CCRDB). The subduction is coloured with red and blue for the sections with high and low coupling respectively. The line with opposing triangles show the sutured zip-type plate contact. The software package GMT^94^ (version 5.4) has been used to produce the figure.

The proposed kinematic model for the North America-Cocos-Caribbean triple junction is shown in Fig. 7. The extraction of the Chortis block with respect to North America and the forearc generates the closing of the zipper whose two branches are the Motagua - Polochic - Swan Island Transform and the Central American volcanic arc shear zone. These two branches join the suture of the Tonalá fault in Chiapas. This eastward shift of the triple junction implies a progressive increase of the coupled zone of the subduction interface from the Gulf of Tehuantepec to Guatemala. The western portion of the forearc is gradually incorporated to the North American plate, and consequently the motion of the forearc is gradually rotated from northwestward directed to westward directed; increasing the subduction interface coupling (Figs 2b and 7).

The available GPS, seismic, topographic and geologic data from bibliography are consistent with a closing zipper-type triple junction. Although we have depicted this junction in Fig. 7 as a pure closing zipper for the sake of simplicity, some left-lateral strike-slip motion can be absorbed in the area of Chiapas^21,22,84,91^, forming a diffuse sinistral closing zipper^86^.

The displacement of the forearc is produced in a similar way to a push-pull train. The North American plate motion towards the west generates the closing of the zipper and the pinning of the forearc in Guatemala. As a consequence the North American plate drags (or pulls) the forearc towards the west acting like a head locomotive. The subduction of the Cocos Ridge and the higher subduction interface coupling in the Costa Rica^14–16^, as is reflected in the GPS vectors in Costa Rica and southern Nicaragua, generates a push in the tail of the forearc, acting like a tail locomotive. The joint action of both processes limits the possible internal deformation along the forearc, causing it to behave almost like a rigid block^57^.

## Data Availability

All the data used is freely available from the original sources. The GPS data is obtained from the original references^5,11,12,17,24,56^. The earthquake focal mechanisms are from the Global CMT catalog^59^ available at http://www.globalcmt.org/. The regional topobathymetry used is from the GEBCO_2014 Grid (version 20150318, www.gebco.net). The mean annual precipitation is from the GPCC Climatology Version 2018 at 0.25°^93^ available at https://www.dwd.de/EN/ourservices/gpcc/gpcc.html. The datasets generated during and/or analysed during the current study are available from the corresponding author on reasonable request.

## Supplementary information

Supplementary figure
