# Supplementary material for: Push-pull driving of the Central America Forearc in the context of the Cocos-Caribbean-North America triple junction
Source: Sci Rep. 2019 Aug 1;9:11164. doi: 10.1038/s41598-019-47617-3 (PMC6671955; doi:10.1038/s41598-019-47617-3)
Supplement: Supplementary file 1 — Supplementary figure [file 41598_2019_47617_MOESM1_ESM.pdf]

Push-pull driving of the Central America Forearc in the context of the Cocos-Caribbean-North America triple junction

José A. Álvarez-Gómez*[1], Alejandra Staller Vázquez[2], José J. Martínez-Díaz[1,3], Carolina Canora[4], Jorge Alonso-Henar[1], Juan M. Insua-Arévalo[1], Marta Béjar-Pizarro[5]

1. Department of Geodynamics, Stratigraphy and Paleontology. Faculty of Geology. Complutense University of Madrid. José Antonio Novais, 12. 28040 Madrid, Spain.

2. Dpto. de Ingeniería Topográfica y Cartografía, ETSI Topografía, Geodesia y Cartografía. Universidad Politécnica de Madrid. 28031 Madrid, Spain.

3. IGEO Geosciences Institute. Severo Ochoa, 7. 28040 Madrid, Spain.

4. Department of Geology and Geochemistry; Science Faculty. Universidad Autónoma de Madrid. Francisco Tomas y Valiente, 7, 28049 Madrid, Spain.

5. Geohazards InSAR Laboratory and Modelling Group. Geoscience Research Department. Geological Survey of Spain (IGME). Alenza 1, 28003 Madrid, Spain

*Correspondence to joseantonio.alvarez@ucm.es

Supplementary figure caption.

Unified GPS velocity field in a fixed North America reference frame.

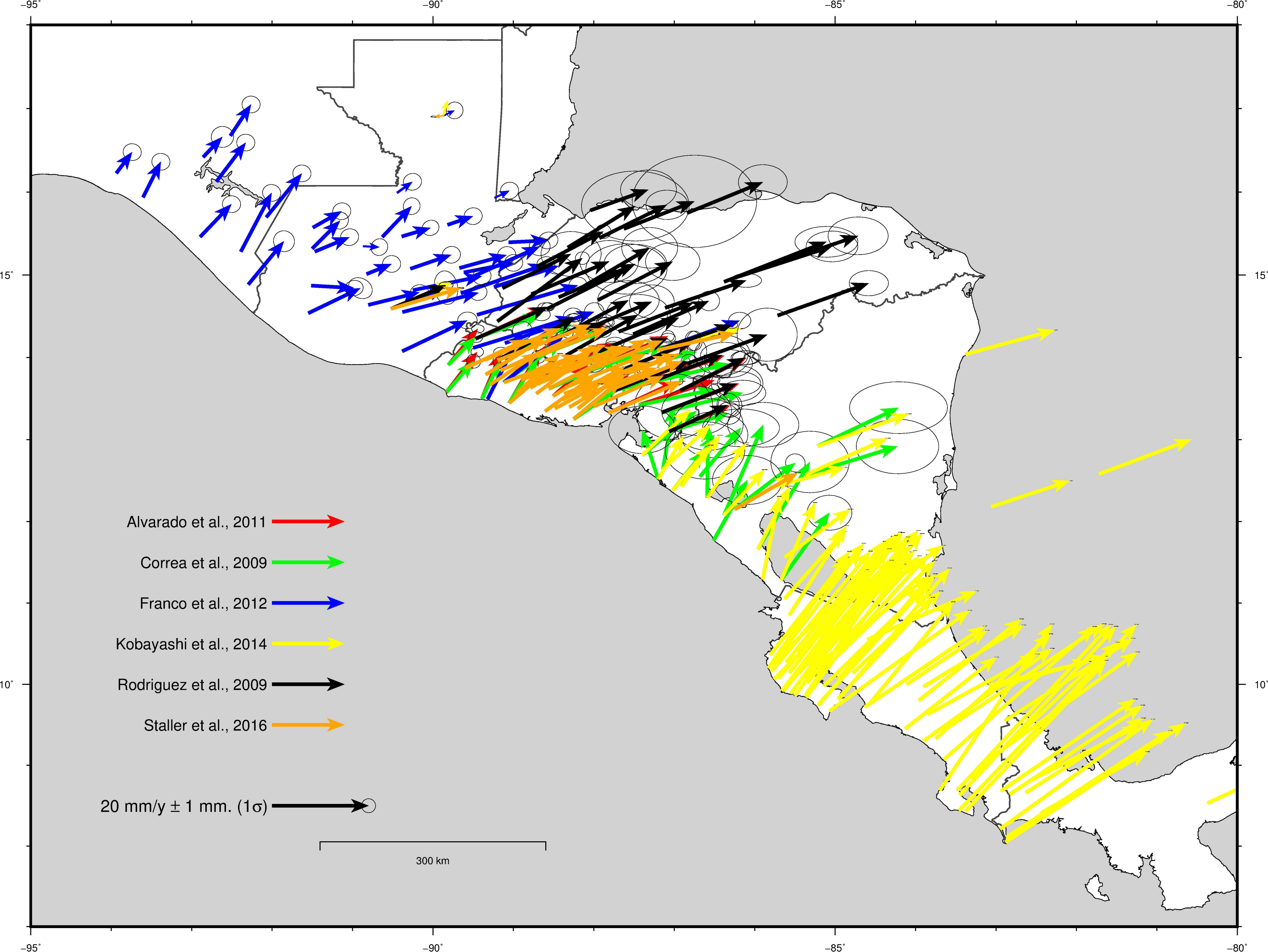

−95˚
−90˚
−85˚
−80˚
15˚
10˚
Alvarado et al., 2011
Correa et al., 2009
Franco et al., 2012
Kobayashi et al., 2014
Rodriguez et al., 2009
Staller et al., 2016
20 mm/y ± 1 mm. (1σ)
300 km
